# Distal Femoral Replacement Following an Intraoperative Periprosthetic Fracture in a Patient with Prior Bone Mulch ACL Reconstruction

**DOI:** 10.1155/2019/6213807

**Published:** 2019-03-31

**Authors:** Anthony Gemayel, Matthew J. Yousif, William Padget, Joseph Finch

**Affiliations:** ^1^Beaumont Hospital Farmington Hills, Farmington Hills, MI, USA; ^2^Beaumont Hospital Dearborn, Dearborn, MI, USA

## Abstract

Periprosthetic distal femur fractures can be treated nonoperatively, with open reduction and internal fixation or with more constrained prostheses. Distal femoral replacement is typically a last resort treatment option for comminuted periprosthetic or osteoporotic distal femoral fractures in patients with poor bone stock or resistant nonunions. We report the case of a 54-year-old female with a remote history of bone mulch ACL reconstruction who sustained an intraoperative comminuted bicondylar distal femur fracture during a primary total knee arthroplasty. This patient was treated with a distal femoral replacement and successfully returned to her preoperative function.

## 1. Introduction

Total knee arthroplasty (TKA) is a common procedure with an estimated 3.4 million TKAs anticipated to be performed annually by 2030 [1]. Several preexisting patient factors may adversely affect the duration, morbidity, and outcome of the procedure. However, one area that remains to be understudied is the effect of a prior anterior cruciate ligament (ACL) reconstruction on a TKA.

**Figure 1:**
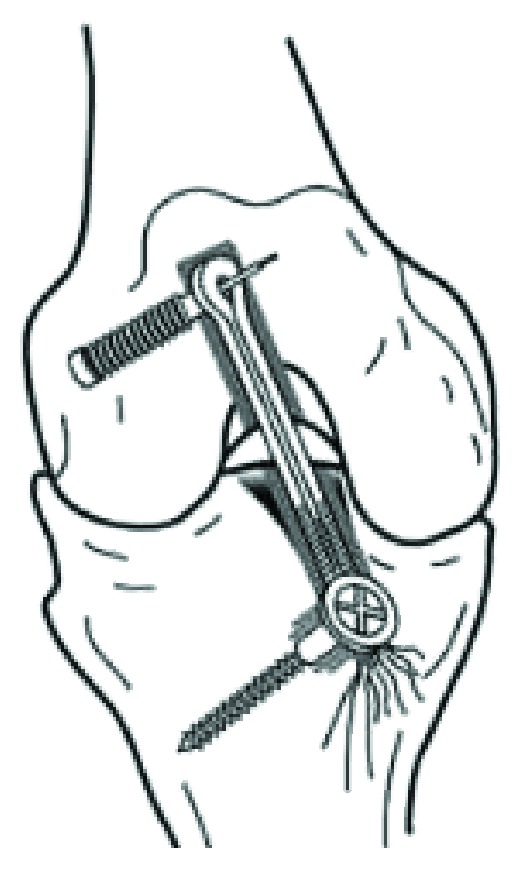
Arthrotek bone mulch ACL screw and WasherLoc.

Approximately 175,000 ACL reconstructions are performed annually [2, 3]. ACL-deficient knees lead to meniscal and chondral damages. While reconstruction improves stability and function, a reduction in the development of secondary osteoarthritis has not been demonstrated [2, 4]. The patient in the case we are presenting underwent ACL reconstruction with the bone mulch screw and WasherLoc from Arthrotek (Figure 1) [5].

A complication which orthopaedic surgeons will inevitably encounter is periprosthetic fractures of the distal femur and tibia. The incidence of periprosthetic fracture ranges from 0.3-2.5%, with the most common location being the supracondylar distal femur; typically, these are due to low-energy trauma [6]. Intraoperative periprosthetic at the time of TKA is rare. Alden et al. found a 0.39% incidence of intraoperative fractures among a sample of 17,389 TKAs. Of these, 73.1% occurred in the femur, most commonly in the medial and lateral condyle [7]. Periprosthetic fractures can occur in the distal femur, proximal tibia, or patella. The most common classification system used for periprosthetic fractures of the distal femur is the Lewis and Rorabeck classification. In this classification system, type I is nondisplaced with a stable component; type II has >5 mm of displacement or >5 degrees of angulation with a stable component; and type III has a loose component [8]. There are several additional classification systems; all of which fail to classify intraoperative periprosthetic fractures. The anatomic location is typically used to describe these fractures. Risk factors that may predispose patients to periprosthetic fractures include anterior notching of the femur, osteoporosis, osteolysis, implant loosening, rheumatoid arthritis, neurologic disorders, corticosteroid use, increased age, and female sex [6, 9, 10].

Various treatment options have been described for periprosthetic fractures of the knee. For nondisplaced fractures with a stable prosthesis, a knee immobilizer or cast can be utilized with protected weight-bearing for 6 weeks postoperatively. Operative treatment may vary depending on the fracture pattern. Treatments that have been described in the literature include external fixation; open reduction internal fixation (ORIF) with a blade plate, submuscular plate, or condylar screw; retrograde intramedullary nail (RIMN); long stem/constrained prosthesis; allograft-prosthetic composite (APC); distal femoral replacement; and arthrodesis [8–15].

## 2. Case

**Figure 2:**
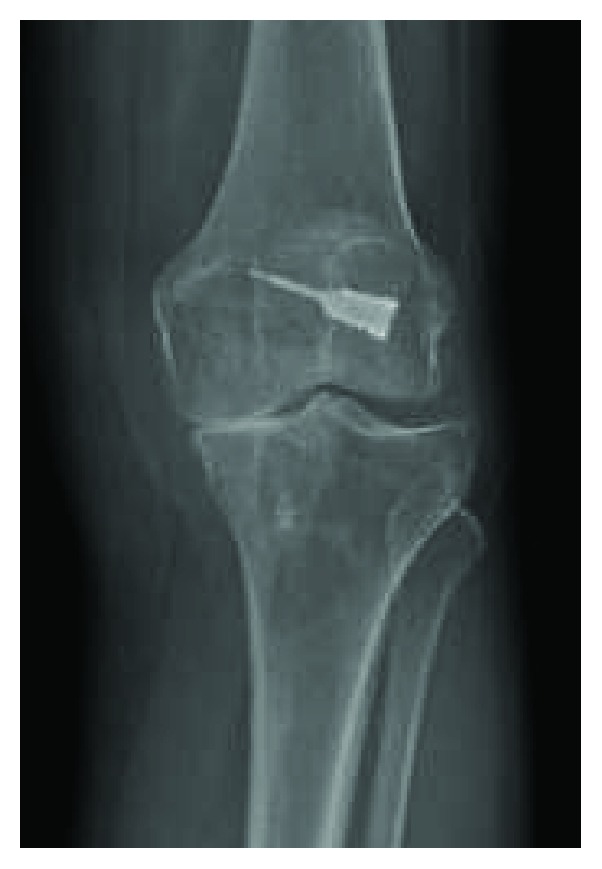
Left knee with significant medial compartmental osteoarthritis and evidence of a previous bone mulch ACL screw.

A 54-year-old Caucasian female with a history of lupus presented for elective left total knee arthroplasty following the development of osteoarthritis that had failed conservative measures. The patient had a history of previous left knee ACL reconstruction approximately 25 years ago using the Arthrotek bone mulch screw and WasherLoc system [5]. She underwent removal of the tibial WasherLoc approximately 10 years later, in the early 2000's (Figure 2).

**Figure 3:**
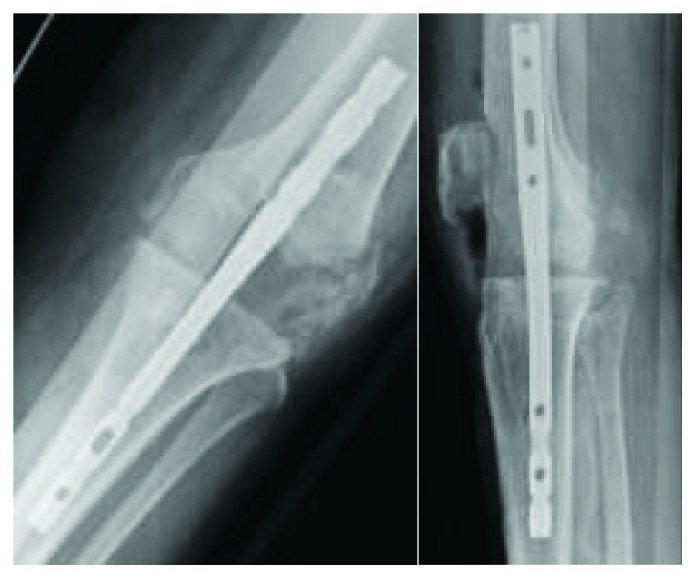
Intraoperative periprosthetic fracture fixated with an intramedullary nail.

**Figure 4:**
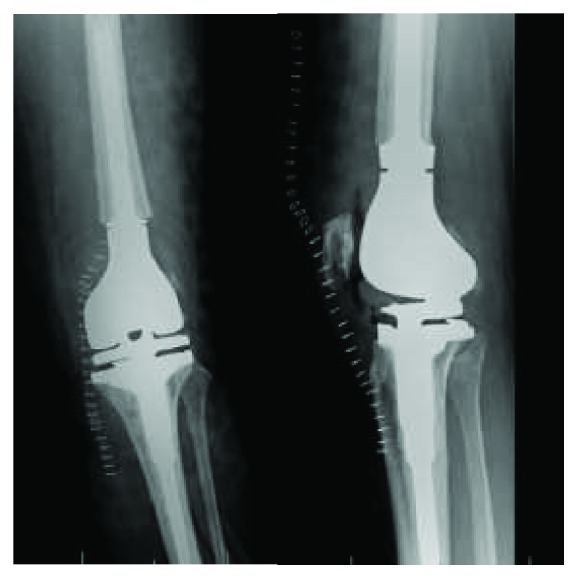
Immediate postoperative radiographs following hinged distal femoral replacement.

The patient was positioned supine; standard incision with a medial parapatellar arthrotomy was performed. A measured resection technique was then performed with an intramedullary guide placed in the femur. The femur was cut in 6 degrees of valgus and 3 degrees of external rotation. A size 4 femoral prosthesis was placed and noted to overhang both medially and laterally on the condyles. At this time, it was decided to downsize the femoral component. The 4 in 1 femoral cutting block was then placed back on the femur and was noted to be in contact with the bone mulch ACL screw. The bone mulch screw was located and identified in the lateral femoral condyle; a curette was used to clear the head of the screw, and it was removed. The proximal tibia was then prepared using an intramedullary guide with 3 degrees of posterior slope. A size 3 tibial component and a 9 mm poly were placed; the knee was noted to be tight in both flexion and extension. An additional 2 mm resection was performed on the proximal tibia. It was noted at this time while trying to trial the prostheses that the lateral femoral condyle was fractured. Conversion to a stemmed femoral component with a cruciate stabilizing prosthesis was attempted. The femoral canal was reamed, and the femoral box cut was made. However, during trialing, the medial femoral condyle was now noted to have a fracture as well. An intraoperative consultation with an adult reconstruction trained orthopaedic surgeon was performed. Immediate surgical correction was not possible due to improper implants being presented. The femoral and tibial canals were then reamed to accept a 200 mm × 9 mm intramedullary nail to act as a temporary internal stabilization device (Figure 3). The knee was irrigated and closed, and the patient was admitted to the floor. The patient was then brought back to the OR on postoperative day 3 following the index procedure. The prior incision was utilized; the wound was copiously irrigated. It was noted that due to the patient's poor bone quality and comminution of the fractures that the only viable option was a distal femoral replacement. The distal femur was resected, the femoral canal was reamed, and a planar was used on the distal femur. A skim cut and reaming of the tibia were performed. The components were trialed. Final implants included a 13 × 127 mm hinged femoral prosthesis and small 1-stemmed tibial tray; a 32 mm patellar component was used, and a size 10 polyethylene was then inserted; all components were cemented. The knee was noted to be stable throughout range of motion with good patellofemoral tracking. The surgical wound was copiously irrigated and closed (Figure 4). Estimated blood loss was 100 mL; no postoperative transfusion was necessary. She was able to bear weight as tolerated immediately postoperatively. The patient's pain was controlled postoperatively, and she worked well with physical therapy and was discharged home with home health care on postoperative day two with 3 weeks of Coumadin for venous thromboembolism prophylaxis.

The first postoperative visit was at two weeks; the patient had some swelling and quadriceps weakness, and the incision was healing well. Range of motion (ROM) was from 0-100°. At 6 weeks, she was still requiring narcotic medication; quadriceps strength was improving, ROM from 0-105°.

At 12 weeks, X-rays remained unchanged; the patient continued to have mild quadriceps weakness and was no longer requiring narcotic medications. ROM was not documented at this visit. At 6 months, ROM was 0-120°. The incision was well healed; X-rays were unchanged. The patient was doing well; however, she continued to have some residual quadriceps weakness and difficulty ambulating long distances. The patient was lost to follow-up after 6 months.

## 3. Discussion

Periprosthetic fractures of the knee are uncommon but are difficult cases to treat. Most cases occur due to low-energy falls; nonetheless, intraoperative fractures can occur, and it is the surgeon responsibility to diligently watch for this during the case. Delasotta et al. found that 50% of periprosthetic fractures occurred during trialing [16]. Both the medial and femoral condyle fractures noted in our case were noted after trialing. Our lateral femoral condyle fracture was noted after trialing a cruciate-retaining prosthesis; whereas Delasotta et al. showed a 0% intraoperative fracture risk with cruciate-retaining implants, although he did find that semiconstrained implants were 9.69 times more likely to lead to an intraoperative fracture than a posterior stabilizing prosthesis [16]. Our medial femoral condyle fracture was noted after attempting to trial a semiconstrained femoral prosthesis. To our knowledge, there are no cases of intraoperative fractures in patients with a previous bone mulch ACL. Several biomechanical studies have shown that the bone mulch ACL screw was able to survive 5,000 cycles and had a significantly higher initial/linear stiffness and a lower rate of slippage than the endobutton and double-looped tendon graft. Yet, it was significantly lower than the patellar tendon graft and interference screw. Higher stiffness fixation devices secure the graft, allow tendon tunnel healing, and restore stability while allowing aggressive rehab [17, 18]. Leroux et al. demonstrated that patients who underwent an ACL reconstruction versus a matched control cohort were seven times more likely to undergo TKA [19]. Prior studies have noted that patients with a history of ACL reconstruction and a subsequent TKA experience 9-15 minutes of increased operative time; however, there was no difference in EBL or postoperative complications [2]. Watters et al. performed a retrospective cohort study of 122 patients with a history of ACL reconstruction and a matched control cohort. They found the ACL reconstruction group to have a statistically significant risk of infection, in 3.3% of the ACL reconstruction group, and a 5.5 times relative risk of reoperation [20]. Magnussen et al. demonstrated that there were no differences in final range of motion, outcome scores, or alignment at the 3-year follow-up. However, they did note that tibial exposure was more difficult with 14% of patients requiring a tibial tubercle osteotomy. 23% of the patients required manipulation under anesthesia due to refractory stiffness, which was corrected [4]. On one case study, we were able to locate discussed a supracondylar distal femur fracture above a TKA at the site of a previous retained ACL femoral staple [21]. Yet, it should be noted that this case was due to a fall 8 years after the TKA and was not an intraoperative complication like ours. Technical errors that can lead to intraoperative fractures include improper bone cuts, aggressive impaction during impaction of a posterior stabilized femoral component, and eccentrically placing a trial component [8]. We were unable to find any other literature describing complications related to the bone mulch ACL screw. Our patient had other risk factors such as being a female, having a history of rheumatoid arthritis, and corticosteroid use, but it was not until the bone mulch screw was removed intraoperatively that the fracture occurred.

As previously mentioned, treatment for periprosthetic fractures includes internal versus external fixation, prostheses with increasing constraint, and stemmed options. External fixation has largely been abandoned as a treatment option due to poor outcomes with pin tract infections and the potential for deep infection [15]. ORIF is a common and successful treatment option. ORIF is associated with a higher nonunion rate than RIMN, while RIMN is associated with a higher rate of malunion [13]. When components are loose, a revision must be performed to a stemmed component. However, when poor bone stock is present or if there is a highly comminuted fracture, a distal femoral replacement may need to be performed. Distal femoral replacement should be reserved as a last resort due to the significant amount of bone that must be removed, uncertain implant longevity, and few bailout treatment options that remain [9, 10, 14]. Kim et al. report that these implants should not be used in patients who are young and active, even if there is poor bone stock due to the highly likelihood of early failure and few alternative treatment options [8]. Our patient has been lost to follow-up; however, to our knowledge, she has not undergone any revision since her distal femoral replacement. Lundh et al. found that their 17 patients who underwent megaprostheses of the knee or hip for fractures had 94% implant survival at 44 months [14]. Several shortcomings of our case include no follow-up data after 6 months and no pain analog or functional score data being collected, as well as our small sample size. Mortazavi et al. evaluated 20 patients (22 knees) that underwent distal femoral replacement. Of those, they obtained preoperative clinical knee society scores (KSS) of 71.8 and functional KSS scores of 42.7 on 10 patients. Postoperatively, the 16 patients followed up long-term had a mean clinical score of 82.8 and mean functional score of 40. They did note a high complication rate with 8 postoperative complications that did not require surgical intervention. Five patients required repeat surgical intervention. They did not note any deep infections [10]. Discretion should be used when using these implants as they have a high complication rate and a relatively high failure rate due to infection and aseptic loosening. Even in the face of poor bone stock, young and active patients should have other forms of reconstruction attempted first [8, 10]. The patient noted in our case was relatively young; however, there were no other salvageable treatments noted intraoperatively. She had significant improvement of her pain and function postoperatively with good function. In the indicated cases, distal femoral replacement is a viable treatment option for periprosthetic fractures.

## 4. Conclusion

Periprosthetic fractures are difficult to treat and may complicate attempted total knee arthroplasty in patients with a previous ACL reconstruction. Distal femoral replacement is a viable option for treatment in comminuted bicondylar distal femoral fractures in patients with poor bone stock.
